# Gender differences in risk factors for high plasma homocysteine levels based on a retrospective checkup cohort using a generalized estimating equation analysis

**DOI:** 10.1186/s12944-021-01459-z

**Published:** 2021-04-12

**Authors:** Jing Zhao, Zhihua Li, Chengbei Hou, Fei Sun, Jing Dong, Xi Chu, Yansu Guo

**Affiliations:** 1 Health Management Department, Xuanwu Hospital, Capital Medical University, No. 45 Changchun Street, Xicheng District, Beijing, 100053 China; 2 Information Center, Xuanwu Hospital, Capital Medical University, Beijing, China; 3 Department of Evidence-based Medicine, Xuanwu Hospital, Capital Medical University, Beijing, China; 4 Beijing Geriatric Healthcare Center, Xuanwu Hospital, Capital Medical University, No. 45 Changchun Street, Xicheng District, Beijing, 100053 China

**Keywords:** Homocysteine, Hyperhomocysteinemia, Aging, Gender difference, Risk factor, Body mass index, Triglycerides

## Abstract

**Background:**

Hyperhomocysteinemia (HHcy) is associated with various health problems, but less is known about the gender differences in risk factors for high plasma homocysteine (Hcy) levels.

**Methods:**

In this study, a retrospective study was carried out on 14,911 participants (7838 males and 7073 females) aged 16–102 years who underwent routine checkups between January 2012 and December 2017 in the Health Management Department of Xuanwu Hospital, China. Anthropometric measurements, including body mass index (BMI) and waist-to-hip ratio, were collected. Fasting blood samples were collected to measure the biochemical indexes. The outcome variable was Hcy level, and a generalized estimating equation (GEE) analysis was used to identify the associations of interest based on gender.

**Results:**

Males exhibited increased Hcy levels (16.37 ± 9.66 vs 11.22 ± 4.76 μmol/L) and prevalence of HHcy (37.0% vs 11.3%) compared with females. Hcy levels and HHcy prevalence increased with age in both genders, except for the 16- to 29-year-old group. GEE analysis indicated that irrespective of gender, aspartate aminotransferase, creatinine, uric acid, low-density lipoprotein cholesterol and high-density lipoprotein cholesterol levels were positively correlated with Hcy levels, and alanine aminotransferase, total cholesterol and glucose were negatively correlated with Hcy levels. However, age, BMI and triglycerides (TGs) were positively correlated with Hcy levels exclusively in females.

**Conclusions:**

Gender differences in risk factors for high plasma Hcy levels were noted. Although common correlational factors existed in both genders, age, BMI and TGs were independent risk factors for Hcy levels specifically in females.

## Background

Homocysteine (Hcy) is a nonconstitutive, thiol-containing amino acid derived from methionine, and folic acid and vitamins B6 and B12 are required for its degradation. Nutrition deficiency, a defective degradation pathway, a methionine-rich diet and decreased renal function can cause elevated plasma Hcy, which is clinically referred to as hyperhomocysteinemia (HHcy) [1]. HHcy is related to many age-associated diseases [2], including cerebral small vessel disease [3, 4], ischemic stroke [5], cognitive impairment disorders [6], chronic kidney disease [7], and cardiovascular and all-cause death [8]. One of the most critical pathogenic roles of HHcy is causing the vascular endothelial dysfunction, the mechanism of which remains incompletely understood [9, 10]. Although folate intervention was proven effective in reducing the elevated Hcy to normal levels in some HHcy patients [11], studies of risk factors for HHcy are urgent and necessary for the early prevention, detection and intervention of HHcy and its related diseases [12].

The prevalence of HHcy was previously explored and found to vary geographically; however, more studies have focused on the associations between HHcy and specific diseases [5, 6, 8, 13]. Different HHcy prevalences reported in different studies could be due to the various population inclusion criteria and different cutoff values adopted and may be affected by ethnicity, genetic factors, and lifestyle behaviors [14, 15]. A meta-analysis by Yang and coworkers reported that the overall prevalence of HHcy in China was 27.5%, indicating that HHcy has become an important public health issue worthy of greater concern [16]. Moreover, the prevalence of HHcy was considerably increased in Chinese elderly individuals [17]. A recent study conducted in the southern region of China revealed a considerably increased overall prevalence of HHcy (50.8%) in routine checkup populations, and related risk factors for HHcy, including gender, age, uric acid (UA), and blood lipids, were identified [18]. However, data on the gender-specific differences in risk factors for high plasma Hcy levels remain limited. Moreover, since many risk analyses for Hcy were actually based on cross-sectional studies [19, 20], retrospective, repeated measurement data from populations undergoing regular checkups may provide more information and yield unexpected results.

This study investigated the risk factors for high plasma Hcy levels based on a 6-year retrospective checkup cohort. Common and gender-specific associations between Hcy level and variables of interest were analyzed using a generalized estimating equation (GEE).

## Methods

### Participants

A retrospective study was performed on 14,911 individuals (7838 males, 7073 females) aged from 16 to 102 years old who underwent routine medical checkups in Xuanwu Hospital, Capital Medical University, China between January 2012 and December 2017. All participants recruited for this study had measurement data on following indexes: body mass index (BMI), waist circumference (WC), hip circumference (HC), waist to hip ratio (WHR), alanine aminotransferase (ALT), aspartate aminotransferase (AST), creatinine (CREA), glucose (Glu), UA, triglycerides (TGs), total cholesterol (TC), low density lipoprotein-cholesterol (LDL-C), high density lipoprotein-cholesterol (HDL-C) and Hcy. During the study period of 2012–2017, the participants started their checkups in different years. If the participant had more than one checkup within a year, only the first checkup data of this year were used. If the participant had checkups in different years, the data of the first checkups in each year were recognized as repeated measurements. Altogether, 28,397 checkups from the 14,911 participants were included. The study complied with the World Medical Association Declaration of Helsinki regarding ethical conduct of research involving human subjects and was approved by the Ethics Committee of Xuanwu Hospital, Capital Medical University, China.

### Measurement

Baseline data and anthropometric measurements were collected by trained staff from the Health Management Department, Xuanwu Hospital. Fasting (≥ 8 h) blood samples were collected for biochemical analysis. Plasma Hcy levels were analyzed using the enzymatic cycling method, and all the other blood biochemical parameters investigated were measured by standard blood biochemistry assays on a HI-TACHI 7600 automated biochemical analyzer (Hi-Tachi, Tokyo, Japan) at Xuanwu Hospital, Capital Medical University.

### Diagnostic criteria

The diagnostic criterion for HHcy was defined as > 15 μmol/L [16]. HHcy was further classified into 3 groups: mild HHcy (15 < Hcy ≤ 30 μmol/L), moderate HHcy (30 < Hcy < 100 μmol/L), and severe HHcy (≥100 μmol/L) [21]. Four BMI categories, including lean, normal, overweight, and obese, were defined as < 18.5 kg/m^2^, 18.5–23.9 kg/m^2^, 24.0–27.9 kg/m^2^, and ≥ 28 kg/m^2^, respectively, and abnormal WHR was defined as ≥0.90 in males and ≥ 0.85 in females. High Glu was defined as ≥6.1 mmol/L. Hyperlipidemia was defined by the presence of any of the following conditions: TG ≥ 2.26 mmol/L, TC ≥ 6.22 mmol/L, LDL-C ≥ 4.14 mmol/L, or HDL-C < 1.04 mmol/L [22]. High ALT and high AST were defined as ALT> 40 IU/L and AST > 40 IU/L, respectively. High CREA was defined as > 99 μmol/L in males and > 81 μmol/L in females [23]. Hyperuricemia was defined as serum UA > 416 μmol/L in males and > 357 μmol/L in females [24].

### Statistical methods

A two-group mean comparison t-test was used to compare the difference between males and females. Prevalence rates were used to describe trends of biochemical variables in each group. ANOVA was used to compare the difference in Hcy means among age groups, and least significant difference (LSD) was used for pairwise comparisons. The chi-square test was applied to compare the prevalence of Hcy among age groups.

To evaluate the influence of the variables on the Hcy level over the 6-year period, GEEs were constructed. To evaluate the fit of the model to the data, the corrected quasi-likelihood under independence model criterion (QICC) was used for GEE analyses. Three structures of spatial working correlation matrices were used during modeling: independent, autoregressive and unstructured. The independent model with the best goodness-of-fit was selected. A value of *P* < 0.05 was considered significant. The statistical analyses were performed using SPSS 23.0 software.

## Results

### Baseline characteristics of the participants

Of the 14,911 participants, 52.57% (*n* = 7838) were male, and 47.43% (*n* = 7073) were female. The average age at the first checkup during the study period was 52.41 ± 14.80 years of age. The baseline characteristics of the cohort members are listed in Table 1. The overall plasma Hcy level in 14,911 participants was 13.92 ± 8.15 μmol/L. Males had considerably increased Hcy level (16.37 ± 9.66 μmol/L) and prevalence of HHcy (37.0%) compared with females (11.22 ± 4.76 μmol/L, 11.3%). Except for LDL-C, significant differences (*P* < 0.01) existed in BMI (25.80 ± 3.33 kg/m^2^ vs 24.09 ± 3.62 kg/m^2^), WHR (0.89 ± 0.06 vs 0.80 ± 0.06), ALT (26.11 ± 20.27 IU/L vs 18.55 ± 13.34 IU/L), AST (23.27 ± 10.94 IU/L vs 21.19 ± 8.08 IU/L), CREA (75.36 ± 17.83 μmol/L vs 55.58 ± 10.74 μmol/L), UA (363.21 ± 83.64 μmol/L vs 265.48 ± 67.60 μmol/L), TG (1.88 ± 1.72 mmol/L vs 1.39 ± 1.00 mmol/L), TC (4.79 ± 0.92 mmol/L vs 4.92 ± 0.96 mmol/L), HDL-C (1.39 ± 0.35 mmol/L vs 1.63 ± 0.38 mmol/L) and Glu (5.75 ± 1.53 mmol/L vs 5.41 ± 1.24 mmol/L) levels between males and females (Table 1). In view of the pivotal role of gender on the Hcy level, the following results were analyzed for each gender separately.

**Table 1:** Baseline characteristics of the cohort participants

Characteristics	Overall (***n*** = 14,911)	Male (***n*** = 7838)	Female (***n*** = 7073)	***P*** value
**Age** (years)	52.41 ± 14.80	53.11 ± 15.48	51.63 ± 13.97	< 0.01
**BMI** (kg/ m^2^)	24.99 ± 3.58	25.80 ± 3.33	24.09 ± 3.62	< 0.01
Lean	362 (2.4%)	95 (1.2%)	267 (3.8%)	
Overweight	6093 (40.9%)	3759 (48.0%)	2334 (33.0%)	
Obesity	2841 (19.1%)	1830 (23.3%)	1011 (14.3%)	
**WHR**	0.85 ± 0.07	0.89 ± 0.06	0.80 ± 0.06	< 0.01
High	6292 (42.4%)	2923 (37.3%)	3369 (47.6%)	
**ALT** (IU/L)	22.52 ± 17.74	26.11 ± 20.27	18.55 ± 13.34	< 0.01
High	1322 (8.9%)	969 (12.4%)	353 (5.0%)	
**AST** (IU/L)	22.29 ± 9.74	23.27 ± 10.94	21.19 ± 8.08	< 0.01
High	456 (3.1%)	297 (3.8%)	159 (2.2%)	
**CREA** (μmol/L)	65.98 ± 17.87	75.36 ± 17.83	55.58 ± 10.74	< 0.01
High	381 (2.6%)	303 (3.9%)	78 (1.1%)	
**UA** (μmol/L)	316.85 ± 90.69	363.21 ± 83.64	265.48 ± 67.60	< 0.01
High	2535 (17.0%)	1857 (23.7%)	678 (9.6%)	
**Hcy** (μmol/L)	13.92 ± 8.15	16.37 ± 9.66	11.22 ± 4.76	< 0.01
High	3698 (24.8%)	2901 (37.0%)	797 (11.3%)	
**TG** (mmol/L)	1.65 ± 1.44	1.88 ± 1.72	1.39 ± 1.00	< 0.01
High	2657 (17.8%)	1828 (23.3%)	829 (11.7%)	
**TC** (mmol/L)	4.85 ± 0.94	4.79 ± 0.92	4.92 ± 0.96	< 0.01
High	1118 (7.5%)	481 (6.1%)	637 (9.0%)	
**LDL-C** (mmol/L)	2.97 ± 0.83	2.96 ± 0.80	2.98 ± 0.85	0.207
High	1199 (8.0%)	559 (7.1%)	640 (9.0%)	
**HDL-C** (mmol/L)	1.50 ± 0.38	1.39 ± 0.35	1.63 ± 0.38	< 0.01
Low	1310 (8.8%)	1029 (13.1%)	281 (4.0%)	
**Glu** (mmol/L)	5.59 ± 1.41	5.75 ± 1.53	5.41 ± 1.24	< 0.01
High	2458 (16.5%)	1619 (20.7%)	839 (11.9%)	

*Abbreviations*: *BMI* body mass index, *WHR* waist to hip ratio, *ALT* alanine aminotransferase, *AST* aspartate aminotransferase, *CREA* creatinine, *UA* uric acid, *Hcy* homocysteine, *TG* total triglyceride, *TC* total cholesterol, *LDL-C* low density lipoprotein- cholesterol, *HDL-C* high density lipoprotein-cholesterol, *Glu* glucose

### Plasma Hcy level and HHcy prevalence based on age and gender

**Fig. 1:**
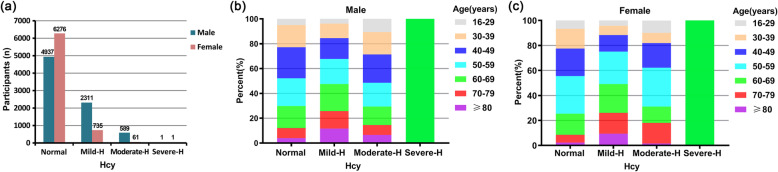
Distribution of Hcy levels in the cohort population and age-group distribution profiles in different groups of plasma Hcy levels. **a** Number of participants, **b** Age-group distribution of males, **c** Age-group distribution of females in different Hcy level groups, including normal (≤15 μmol/L), mild HHcy (15 < Hcy ≤ 30 μmol/L), moderate HHcy (30 < Hcy < 100 μmol/L), and severe HHcy (≥100 μmol/L). Hcy, homocysteine; HHcy, hyperhomocysteinemia; Mild-H, mild HHcy; Moderate-H, moderate HHcy; Severe-H, severe HHcy

**Fig. 2:**
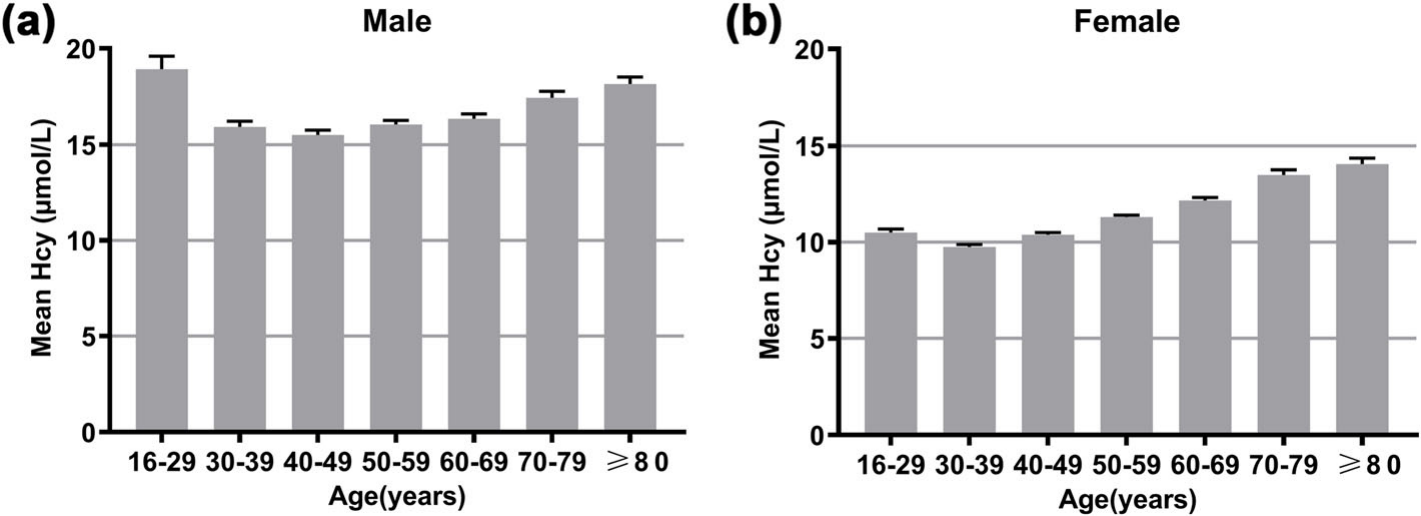
Mean Hcy levels in different age groups (16–29, 30–39, 40–49, 50–59, 60–69, 70–79, and ≥ 80 years of age) in males (**a**) and females (**b**). Error bar stands for standard error. Significant differences are found between 16 and 29 and 30–39 (*P* < 0.01), 16–29 and 40–49 (*P* < 0.01), 16–29 and 50–59 (*P* < 0.01), 16–29 and 60–69 (*P* < 0.01), 16–29 and 70–79 (*P* < 0.05), 30–39 and 70–79 (*P* < 0.01), 30–39 and ≥ 80 (*P* < 0.01), 40–49 and 60–69 (*P* < 0.05), 40–49 and 70–79 (*P* < 0.01), 40–49 and ≥ 80 (*P* < 0.01), 50–59 and 70–79 (*P* < 0.01), 50–59 and ≥ 80 (*P* < 0.01), 60–69 and 70–79 (*P* < 0.05), and 60–69 and ≥ 80 (*P* < 0.01) age groups in males (**a**). However, in females, significant differences exist in any two groups (*P* < 0.01) except that there are no statistical differences between 16 and 29 and 40–49, and 70–79 and ≥ 80 age groups (*P* > 0.05) (**b**). Hcy, homocysteine

**Fig. 3:**
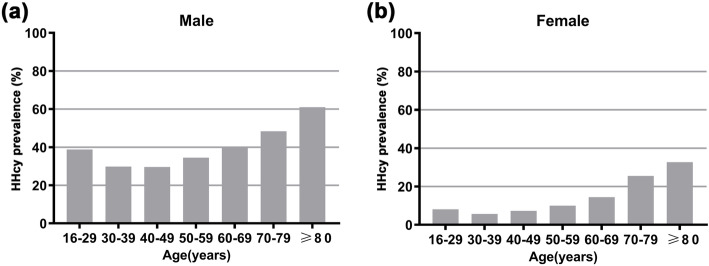
HHcy prevalence in different age groups (16–29, 30–39, 40–49, 50–59, 60–69, 70–79, and ≥ 80 years of age) in males (**a**) and females (**b**). Significant differences in HHcy prevalence are found in any two groups (*P* < 0.01) except that there are no statistical differences between 16 and 29 and 50–59, 16–29 and 60–69, and 30–39 and 40–49 age groups in males (*P* > 0.05). Whereas in females, except that no statistical differences are observed between 16 and 29 and 30–39, 16–29 and 40–49, 16–29 and 50–59, and 30–39 and 40–49 age groups (*P* > 0.05), significant differences exist between any other two groups (*P* < 0.05 between 70 and 79 and ≥ 80, *P* < 0.01 between any other pairs). HHcy, hyperhomocysteinemia

All participants were classified into seven age groups (16–29, 30–39, 40–49, 50–59, 60–69, 70–79, and ≥ 80 years of age) based on gender. The distribution profile of plasma Hcy levels in males and females is shown in Fig. 1a. The ratio of females with normal Hcy levels (88.73%, *n* = 6276) was greater than that of males (62.99%, *n* = 4937). However, the ratio of mild HHcy and moderate HHcy in males 29.48%, *n* = 2311; and 7.51%, *n* = 589) was considerably increased compared with that in females (10.39%, *n* = 735; and 0.86%, *n* = 61). There were only 2 participants with severe HHcy in this study, including 1 male (0.01%) and 1 female (0.01%). The age distribution in each Hcy-based group is shown in Fig. 1b, c. Regarding the average Hcy levels in each age group, males had higher mean plasma Hcy levels than females in all of the age groups (Fig. 2). There was an ascending trend in the mean Hcy level with increased age in both males and females. Surprisingly, the 16- to 29-year-old group had a noticeably higher mean Hcy level than the adjacent 30- to 39-year-old group (*P* < 0.01, Fig. 2a, b). In males, the average Hcy level of the 16- to 29-year-old group was approximately equivalent to that of the ≥80-year-old group (*P* > 0.05, Fig. 2a). Similarly, the HHcy prevalence in each age group in males was greater than that in the corresponding age group in females (Fig. 3). The HHcy prevalence increased with age in both genders with the exception that the 16- to 29-year-old group in males had a remarkably greater HHcy prevalence compared with both the 30–39 and 40–49 year groups (*P* < 0.01, Fig. 3a).

### Risk factor analysis for high plasma Hcy levels based on gender

Associations between Hcy level and variables of interest in males are presented in Table 2. After adjustment for confounders, the Hcy level was associated with increases in AST (B = 0.079, *P* < 0.01), CREA (B = 0.082, *P* < 0.01), UA (B = 0.005, *P* < 0.01), LDL-C (B = 0.575, *P* = 0.03) and HDL-C (B = 0.911, *P* < 0.01) and decreases in ALT (B = -0.052, *P* < 0.01), TC (B = -0.621, *P* = 0.014) and Glu (B = -0.239, *P* < 0.01). Age, BMI, WHR and TGs were not associated with Hcy levels in males (Table 2).

**Table 2:** Generalized estimating equation analysis on the association between Hcy level and variables of interest over 6 year period in males

variables	B	95% Confidence Interval		***P*** value
		Lower	Upper	
**Age**	−0.004	−0.020	0.012	0.614
**BMI**	−0.014	−0.090	0.061	0.710
**WHR**	4.220	−0.045	8.485	0.052
**ALT**	**−0.052**	**− 0.072**	**− 0.032**	**< 0.01**
**AST**	**0.079**	**0.039**	**0.118**	**< 0.01**
**CREA**	**0.082**	**0.072**	**0.093**	**< 0.01**
**UA**	**0.005**	**0.002**	**0.008**	**< 0.01**
**TG**	0.141	−0.028	0.310	0.101
**TC**	**−0.621**	**−1.116**	**−0.125**	**0.014**
**LDL-C**	**0.575**	**0.055**	**1.094**	**0.030**
**HDL-C**	**0.911**	**0.245**	**1.576**	**< 0.01**
**Glu**	**−0.239**	**−0.344**	**−0.134**	**< 0.01**

*Abbreviations*: *Hcy* homocysteine, *BMI* body mass index, *WHR* waist to hip ratio, *ALT* alanine aminotransferase, *AST* aspartate aminotransferase, *CREA* creatinine, *UA* uric acid, *TG* total triglyceride, *TC* total cholesterol, *LDL-C* low density lipoprotein- cholesterol, *HDL-C* high density lipoprotein-cholesterol, *Glu* glucose

In females, all of the variables of interest except for WHR were significantly associated with Hcy levels (Table 3). Hcy levels were correlated with increases in age (B = 0.051, *P* < 0.01), BMI (B = 0.094, *P* < 0.01), AST (B = 0.063, *P* < 0.01), CREA (B = 0.076, *P* < 0.01), UA (B = 0.003, *P* < 0.01), TG (B = 0.178, *P* < 0.01), LDL-C (B = 0.633, *P* < 0.01) and HDL-C (B = 0.628, *P* < 0.01) and reductions in ALT (B = -0.040, *P* < 0.01), TC (B = -0.547, *P* < 0.01) and Glu (B = -0.142, *P* < 0.01). Age, BMI and TGs were independently associated with Hcy levels specifically in females (Table 3).

**Table 3:** Generalized estimating equation analysis on the association between Hcy level and variables of interest over 6 year period in females

Parameter	B	95% Confidence Interval		***P*** value
		Lower	Upper	
**Age**	**0.051**	**0.041**	**0.060**	**< 0.01**
**BMI**	**0.094**	**0.048**	**0.139**	**< 0.01**
**WHR**	0.575	−1.557	2.708	0.597
**ALT**	**−0.040**	**−0.055**	**−0.026**	**< 0.01**
**AST**	**0.063**	**0.038**	**0.089**	**< 0.01**
**CREA**	**0.076**	**0.056**	**0.095**	**< 0.01**
**UA**	**0.003**	**0.001**	**0.005**	**< 0.01**
**TG**	**0.178**	**0.046**	**0.311**	**< 0.01**
**TC**	**−0.547**	**−0.770**	**−0.324**	**< 0.01**
**LDL-C**	**0.633**	**0.395**	**0.871**	**< 0.01**
**HDL-C**	**0.628**	**0.345**	**0.912**	**< 0.01**
**Glu**	**−0.142**	**−0.208**	**− 0.077**	**< 0.01**

*Abbreviations*: *Hcy* homocysteine, *BMI* body mass index, *WHR* waist to hip ratio, *ALT* alanine aminotransferase, *AST* aspartate aminotransferase, *CREA* creatinine, *UA* uric acid, *TG* total triglyceride, *TC* total cholesterol, *LDL-C* low density lipoprotein- cholesterol, *HDL-C* high density lipoprotein-cholesterol, *Glu* glucose

## Discussion

In the present study, an overall Hcy level of (13.92 μmol/L) in 14,911 participants was recognized, which is lower than the level of (15.27 μmol/L) previously reported in the Guangxi checkup population [18]. This study demonstrated that Hcy levels in males are approximately 1.5 times that of females and that HHcy is 3.3-fold more common in males than females. Both plasma Hcy level and HHcy prevalence increase with age, with the exception of the 16–29 year group. More importantly, except for some common associations irrespective of gender, this study revealed that age, BMI and TGs were significantly correlated with Hcy levels in female participants in a 6-year retrospective checkup cohort.

Elevated Hcy levels exhibit a linear association with all-cause mortality. When the Hcy level increases each 5 μmol/L, the risk of all-cause mortality increases by 33.6% [25]. This study found a relatively lower prevalence of HHcy (24.8%) in this cohort compared with an earlier meta-analysis (27.5%) [16]. Consistent with previous studies [17, 18, 26], males exhibit increased Hcy levels (16.37 μmol/L vs 11.22 μmol/L) and a higher prevalence of HHcy (37.0% vs 11.3%) compared with females, and lifestyle factors, such as exposure to environmental cigarette smoking and alcohol consumption [27, 28], genetic variation of methylene tetrahydrofolate reductase (MTHFR) [29], and rates of remethylation and transmethylation of Hcy [30], may contribute to sexual differences. Although gender differences in Hcy levels have been widely noticed, gender is typically used as a variable or influential factor in statistical analyses. Separate gender analyses on the distribution profile of Hcy level, HHcy prevalence and risk factors for high plasma Hcy are noteworthy and essential. Although advanced age was reported to be associated with elevated Hcy levels [31], this study demonstrated an ascending trend of plasma Hcy levels and HHcy prevalence with increased age based on gender in a multiaged population. Of note, the 16- to 29-year-old group exhibited exceptionally increased Hcy levels and HHcy prevalence, especially in males, indicating that more attention should be paid to the young adult group. In a recent study, the conicity index-adjusted total body fat exhibited a closer relationship with HHcy in 20- to 40-year-old adults [32]. All these results suggest that HHcy and metabolic health problems in young adults deserve further attention.

In the present work, to make maximum use of the data, a retrospective checkup cohort was generated, and GEE analysis was applied to examine the correlation factors for Hcy levels in both genders. Among the entire cohort members, 39% had two or more checkups during the 6-year period, and only data from the first checkups with all variables of interest recorded in each year were used. The results showed that Hcy levels were correlated with increases in AST, CREA, UA, LDL-C and HDL-C and declines in ALT, TC and Glu in both males and females. WHR was not correlated with Hcy level in either gender. This result is consistent with the findings of Widiana et al. [33], who showed that CREA clearance was correlated with plasma Hcy in predialytic chronic renal failure patients. In addition, a prospective study of hypertensive subjects without chronic kidney disease showed that HHcy can serve as a biomarker to predict renal function decline [34]. Significantly higher Hcy levels have also been observed in subjects with increased CREA levels [35]. Indeed, UA was reported to be positively related to Hcy concentration and metabolic syndrome in both genders [18, 36]. Although the liver plays a central role in the synthesis and metabolism of Hcy and related thiols, the data reported on the association of plasma Hcy levels with liver damage are conflicting [37, 38]. This study indicated that ALT was a protective factor for Hcy levels in both males and females, supporting the opinion that increased Hcy levels are negatively associated with the histological severity of nonalcoholic fatty liver disease [39]. Moreover, Hcy regulates lipid metabolism [40], and correlations between Hcy levels and lipid profiles were investigated. The results indicate a positive relationship between HDL-C and Hcy levels, which is not consistent with the negative correlation in Momin’s study [19]. In addition, a correlation between Hcy and LDL-C (positive) or TC (negative) was also shown in both genders in this study. Recently, a positive association between Hcy and impaired glucose tolerance was reported [41]. However, the current study indicated that the Hcy level was correlated with a reduction in blood glucose. These results indicate that to some degree, higher glucose levels are a protective factor for high Hcy levels.

It is worth noting that unlike previous studies showing that age, BMI [42], and hypertriglyceridemia [19] were risk factors for HHcy in healthy subjects, this study indicated that plasma Hcy levels were only significantly and positively correlated with age, BMI and TGs in females, indicating the importance of gender-based prevention. Coincidentally, plasma Hcy levels were reported to be correlated with endothelial dysfunction exclusively in female hypertensive patients [43]. In contrast, using multiple logistic regression models, Wang’s study showed that BMI-based general obesity was not related to the risk of HHcy in middle-aged women, whereas WC-based central obesity was [20]. Different study subjects, designs and statistical methods may contribute to the different conclusions. Nevertheless, understanding both common and gender-specific risk factors for Hcy levels may provide important indications for both general and gender-targeted preventions for HHcy and its related diseases.

### Study strength and limitation

This study had several strengths. First, it is a multiaged checkup population with repeated measurement data within a 6-year period. Second, gender differences in risk factors for high Hcy levels were identified. There were also limitations that should be mentioned. First, it was a retrospective study, and many factors, including lifestyle, daily diet, and medication history, which may have an effect on Hcy levels, were not collected. Second, given that the participants in this study were from a single medical center-based checkup population, the characteristics and risk factors concluded from these subjects may differ from those of the general community. Therefore, a large-scale community-based cohort study or multicenter prospective study may be necessary to further validate the results.

## Conclusions

The present study demonstrates that males exhibit increased plasma Hcy levels and HHcy prevalence compared with females in any age group. In addition, young adults under 30 years of age should be considered. Gender-specific differences in risk factors for high Hcy levels exist in Beijing checkup populations. Age, BMI and TGs were independent risk factors correlated with Hcy levels in only females. BMI management and TG control may aid in the prevention of HHcy and related diseases, especially in elderly females.

## Data Availability

The dataset supporting the conclusions of this article is available from the corresponding author upon reasonable request.

## Abbreviations

Hcy: Homocysteine
HHcy: Hyperhomocysteinemia
BMI: Body mass index
GEE: Generalized estimating eq.
WC: Waist circumference
HC: Hip circumference
WHR: Waist-to-hip ratio
ALT: Alanine aminotransferase
AST: Aspartate aminotransferase
CREA: Creatinine
Glu: Glucose
UA: Uric acid
TGs: Triglycerides
TC: Total cholesterol
HDL-C: High-density lipoprotein cholesterol
LDL-C: Low-density lipoprotein cholesterol
LSD: Least significant difference
